# Supplementary material for: Complexome profiling of the Chlamydomonas psb28 mutant reveals TEF5 as an early PSII assembly factor
Source: Plant Cell. 2025 Mar 17;37(6):koaf055. doi: 10.1093/plcell/koaf055 (PMC12164590; doi:10.1093/plcell/koaf055)
Supplement: koaf055_Supplementary_Data [file koaf055_supplementary_data.zip › psb28_interactive.zip!/plots/intensity/qualityPlots/peptides/PHOT1_Cre03.g199000.t1.2.html]

### Cre03.g199000.t1.2

Trivial name: PHOT1  
SeqCoverage [median]: 14.30  
Mass: 81.39 kDa  
  
 

| Sequence | MissedCleavage | Mass [Da] | wtIntensity | muIntensity | Score |
| --- | --- | --- | --- | --- | --- |
| AAIKEGSELTVR | 1 | 1272.70 | 4139.41 | 9977.32 | 112.43 |
| ALADNSGVPLLVK | 0 | 1295.74 | 2249.06 | 7654.84 | 110.38 |
| FLQGAGTDR | 0 | 963.48 | 0.00 | 344.46 | 63.48 |
| HQQPPYVPR | 0 | 1120.58 | 0.00 | 494.81 | 69.60 |
| IGGAGAAGGSAPK | 0 | 1012.53 | 1008.69 | 4150.38 | 77.66 |
| TGANEIK | 0 | 731.38 | 406.81 | 1844.31 | 129.68 |
| TLDKFEMQER | 1 | 1295.62 | 1627.40 | 2287.12 | 59.54 |
| TPEEEVAK | 0 | 901.44 | 243.57 | 0.00 | 98.52 |
| VALDLATTVER | 0 | 1186.66 | 3526.16 | 0.00 | 98.04 |
|  |  |  | 13201.1 | 26753.2 |  |
